# Supplementary material for: Life-course blood pressure trajectories and cardiovascular diseases: A population-based cohort study in China
Source: PLoS One. 2020 Oct 21;15(10):e0240804. doi: 10.1371/journal.pone.0240804 (PMC7577482; doi:10.1371/journal.pone.0240804)
Supplement: S1 Table — Abbreviations: BIC = Bayesian information criterion. *Model in bold font was chosen as the best model and included as the main analysis in the study. (DOCX) [file pone.0240804.s001.docx]

**S1 Table. The parameters of latent class growth models (n=3566)**

| No. Latent classes | Log-likelihood | BIC | % Group membership | Mean Posterior probabilities |
| --- | --- | --- | --- | --- |
| 1 | -91556.97 | 183146.7 | 100 | 1.00 |
| 2 | -89462.62 | 178982.5 | 69.9/30.1 | 0.93 /0.88 |
| 3 | -88860.62 | 177819.4 | 46.4/45.3/8.4 | 0.86/0.83/0.88 |
| 4 | -88638.83 | 177408.5 | 49.1/24.6/21.1/5.1 | 0.76/0.78/0.81/0.88 |
| **5^*^** | **-88476.62** | **177116.8** | **4.3/48.8/20.9/23.7/2.3** | **0.85/0.76/0.82/0.76/0.85** |
| 6 | -88438.34 | 177064.8 | 2.6/24.1/20.8/48.8/0.3/3.5 | 0.86/0.77/0.82/0.77/0.92/0.79 |
| 7 | -88339.05 | 176678.1 | 2.9/21.2/8.5/15.9/43.5/6.4/1.6 | 0.77/0.59/0.67/0.81/0.72/0.67/0.88 |

Abbreviations: BIC=Bayesian information criterion.

^*^Model in bold font was chosen as the best model and included as the main analysis in the study.
