# Supplementary material for: Life-course blood pressure trajectories and cardiovascular diseases: A population-based cohort study in China
Source: PLoS One. 2020 Oct 21;15(10):e0240804. doi: 10.1371/journal.pone.0240804 (PMC7577482; doi:10.1371/journal.pone.0240804)
Supplement: S2 Table — Abbreviations: CVD = cardiovascular disease. *Model 1 was adjusted for socio-demographic factors (i.e., age, sex, living region and education) and baseline SBP, and model 2 was further adjusted for smoking, alcohol overconsumption, physical activity, unhealthy dietary and body mass index. (DOCX) [file pone.0240804.s002.docx]

**S2 Table. The association between systolic blood pressure trajectories and cardiovascular diseases by use of antihypertensive drugs (n=3566)**

|  | **Without use of antihypertensive drugs (n=3068)** | | | | **Using antihypertensive drugs (n=498)** | | | |
| --- | --- | --- | --- | --- | --- | --- | --- | --- |
| **Trajectory group** | **No. of subjects** | **No. of CVD cases** | **Odds ratio (95% confidence interval)^*^** | | **No. of subjects** | **No. of CVD cases** | **Odds ratio (95% confidence interval)^*^** | |
|  |  |  | Model 1 | Model 2 |  |  | Model 1 | Model 2 |
| **Slight increase** | 1828 | 34 | Ref | Ref | 130 | 12 | Ref | Ref |
| **Stable** | 599 | 9 | 0.78 (0.36, 1.70) | 0.81 (0.37, 1.77) | 15 | 2 | 1.35 (0.25, 7.13) | 1.37 (0.25, 7.52) |
| **Increase** | 571 | 11 | 1.04 (0.50, 2.18) | 1.04 (0.49, 2.20) | 229 | 28 | 1.86 (0.88, 3.94) | 1.77 (0.82, 3.82) |
| **Rapid increase** | 45 | 0 | - | - | 68 | 9 | 2.99 (1.10, 8.16) | 2.81 (1.01, 7.77) |
| **Fluctuant** | 25 | 1 | 1.75 (0.20, 15.75) | 1.45 (0.16, 13.04) | 56 | 10 | 3.19 (1.02, 9.98) | 2.94 (0.93, 9.31) |

Abbreviations: CVD=cardiovascular disease.

^*^Model 1 was adjusted for socio-demographic factors (i.e., age, sex, living region and education) and baseline SBP, and model 2 was further adjusted for smoking, alcohol overconsumption, physical activity, unhealthy dietary and body mass index.
