# Supplementary material for: Life-course blood pressure trajectories and cardiovascular diseases: A population-based cohort study in China
Source: PLoS One. 2020 Oct 21;15(10):e0240804. doi: 10.1371/journal.pone.0240804 (PMC7577482; doi:10.1371/journal.pone.0240804)
Supplement: S3 Table — Abbreviations: CVD = cardiovascular disease. *Model 1 was adjusted for socio-demographic factors (i.e., age, sex, living region and education) and baseline SBP, model 2 was further adjusted for smoking, alcohol overconsumption, physical activity, unhealthy dietary and body mass index, and model 3 was additionally adjusted for antihypertensive drugs. (DOCX) [file pone.0240804.s003.docx]

**S3 Table. The association between systolic blood pressure trajectories and cardiovascular diseases among those followed from baseline to last visit (n=2210)**

| **Trajectory group** | **No. of subjects** | **No. of CVD cases** | **Odds ratio (95% confidence interval)^*^** | | |
| --- | --- | --- | --- | --- | --- |
|  |  |  | Model 1 | Model 2 | Model 3 |
| **Slight increase** | 1147 | 32 | Ref | Ref | Ref |
| **Stable** | 421 | 8 | 0.59 (0.26, 1.31) | 0.61 (0.27, 1.37) | 0.75 (0.33, 1.71) |
| **Increase** | 504 | 26 | 2.02 (1.15, 3.52) | 1.98 (1.12, 3.49) | 1.18 (0.63, 2.20) |
| **Rapid increase** | 89 | 7 | 3.87 (1.59, 9.43) | 3.59 (1.44, 8.96) | 1.62 (0.60, 4.35) |
| **Fluctuant** | 49 | 9 | 5.60 (1.92, 16.27) | 5.13 (1.74, 15.13) | 2.36 (0.77, 7.26) |

Abbreviations: CVD=cardiovascular disease.

^*^Model 1 was adjusted for socio-demographic factors (i.e., age, sex, living region and education) and baseline SBP, model 2 was further adjusted for smoking, alcohol overconsumption, physical activity, unhealthy dietary and body mass index, and model 3 was additionally adjusted for antihypertensive drugs.
