# Supplementary material for: Life-course blood pressure trajectories and cardiovascular diseases: A population-based cohort study in China
Source: PLoS One. 2020 Oct 21;15(10):e0240804. doi: 10.1371/journal.pone.0240804 (PMC7577482; doi:10.1371/journal.pone.0240804)
Supplement: Attachment — Submitted filename: review(Huang) 20200612.docx [file pone.0240804.s004.docx]

Dr. Xu and colleagues investigated the association between life-course blood pressure trajectories and cardiovascular diseases in a population based cohort study in China. The major finding is that rapid increase and fluctuant patterns of SBP trajectory over life course are associated with higher risk of CVDs.

1. The study showed that the “fluctuant” group started with a low SBP (~70 mmHg) in childhood, which was not consistent with the common sense, please explain the reason.

2. Tables 2 and S2 showed different results between “Rapid increase” group and “Fluctuant” group, which needed to be further explained.

3. The table did not show the characteristics of the study population across trajectory groups (n=3750), which were analyzed in this study.

4. Did the events include both recurrent and new cases of CVD?
