# Supplementary material for: Life-course blood pressure trajectories and cardiovascular diseases: A population-based cohort study in China
Source: PLoS One. 2020 Oct 21;15(10):e0240804. doi: 10.1371/journal.pone.0240804 (PMC7577482; doi:10.1371/journal.pone.0240804)
Supplement: Attachment — Submitted filename: Response to comments_2020Jul28.docx [file pone.0240804.s005.docx]

Journal Requirements:

1. Please ensure that your manuscript meets PLOS ONE's style requirements, including those for file naming. The PLOS ONE style templates can be found at

<https://journals.plos.org/plosone/s/file?id=wjVg/PLOSOne_formatting_sample_main_body.pdf> and

<https://journals.plos.org/plosone/s/file?id=ba62/PLOSOne_formatting_sample_title_authors_affiliations.pdf>

Answer: We have revised the manuscript according to PLOS ONE style requirements.

2. We note that you have stated that you will provide repository information for your data at acceptance. Should your manuscript be accepted for publication, we will hold it until you provide the relevant accession numbers or DOIs necessary to access your data. If you wish to make changes to your Data Availability statement, please describe these changes in your cover letter and we will update your Data Availability statement to reflect the information you provide.

Answer: The Data Availability statement has been changed and added in the cover letter.

3. We note that you have included the phrase “data not shown” in your manuscript. Unfortunately, this does not meet our data sharing requirements. PLOS does not permit references to inaccessible data. We require that authors provide all relevant data within the paper, Supporting Information files, or in an acceptable, public repository. Please add a citation to support this phrase or upload the data that corresponds with these findings to a stable repository (such as Figshare or Dryad) and provide and URLs, DOIs, or accession numbers that may be used to access these data. Or, if the data are not a core part of the research being presented in your study, we ask that you remove the phrase that refers to these data.

Answer: We have removed the phrase “data not shown”. All of the results are now shown in the revised manuscript.

4. We note you have included a table to which you do not refer in the text of your manuscript. Please ensure that you refer to Table 1 in your text; if accepted, production will need this reference to link the reader to the Table.

Answer: We apologize for the carelessness. This has been revised and all figure and tables are now referred in the text.

5. Please include captions for your Supporting Information files at the end of your manuscript, and update any in-text citations to match accordingly. Please see our Supporting Information guidelines for more information: [http://journals.plos.org/plosone/s/supporting-information](about:blank).

Answer: We have added the captions for the supporting materials after references, see pages 20-21, lines 400-403.

**Reviewer #1:**

Major comments:

1. The SBP trajectories cannot be deemed as lifelong SBP trajectories. The follow-up duration is shorter compared to the whole lifespan and thus heterogenous participants, the youth and the elderly, are likely to be modelled into the same trajectory. Moreover 95% of total participants aged 17-47 years (Table 1). Modelling within this age range may be appropriate whereas modelling out of the range misleading. A clear trend toward persistent high blood pressure of the "fluctuant" group when compared to other groups is observed within this age range, which is consistent with our common sense that high blood pressure, but not "fluctuant" SBP, increases CV risk.

Answer: The age range of study participants was 9-70 years old at baseline. Thus, the 20-year’s SBP trajectory covered the age from 9 years old (youngest at baseline) to 90 years (oldest at the end of follow-up). The modelling of the trajectory was within this age range, which can be considered from a lifetime perspective. We agree that the trajectory in childhood and late life are less reliable due to the small number of participants. We have acknowledged this as a limitation in the revised version (see page 15, lines 274-275).

2. LCGM modelling strategy should be re-evaluated. It takes at least three repeated measures to conduct trajectory analysis, thus participants with 1-2 SBP readings should be excluded in the first place. Besides, each trajectory is expected to have at least 5% of the overall population. In the current analysis, "rapid increase" group and "fluctuant" group both comprise 2% of total participants, which make it hard to interpret at clinical perspective. Mean posterior probabilities are recommended at > 0.7-0.8, why did the authors choose 0.65 as the cutoff? In addition, the analysis of SBP trajectory and CV events is restricted to 3750 participants (73%), thus the BP trajectories should be re-modelled.

Answer: We thank the reviewer for these suggestions. We have re-modelled the trajectory analysis among those with at least 3 SBP measurements (n=3600). Accordingly, the description and results in both abstract and main text have been revised (see pages 2,4,5).

In light of these analyses, we still choose the model with 5 trajectory classes as the final model. The mean posterior probability for each class in the updated model meets the recommended criteria (0.76-0.85). The percentage is still small for two groups: 2.3% for fluctuant group, and 3.1% for rapid increase group. We agree with the reviewer that each class is expected to have at least 5% of the participants, which is good to have enough power for the subsequent analysis. However, this is not a necessary requirement for a model decision as done in the previous studies (Buscot M-J, et al. Distinct child-to-adult body mass index trajectories are associated with different levels of adult cardiometabolic risk. Eur Heart J 2018; 39:2263-2270.). We acknowledged that with the small percent of some group (e.g., 3.1% for rapid increase), we have a reduced power for the subsequent associations between SBP trajectory and CVDs. We have acknowledged this as a limitation (see page 15, lines 275-278).

We know that statistical parameters (e.g., BIC, group membership, mean posterior probability) are very important for the model chosen, but this cannot be considered as the only criteria in the trajectory analysis. We have chosen the model also based on the assumption of SBP change over life course (e.g., distinct change patterns including fluctuant variations). If the model is chosen solely on the statistical parameters, we may not be able to identify those with fluctuant SBP levels, which do exist in reality and is informative from a clinical perspective. To make it more clear, we have modified the description in the statistical analysis (see page 7, lines 149-153).

3. Since baseline SBP level is a strong predictor of prospective CV events and the authors had emphasized in the introduction that BP trajectory should be considered as an auxiliary factor to BP in CV risk evaluation, baseline SBP should be adjusted in the logistic model.

Answer: As the reviewer suggested, we have adjusted baseline SBP in the association between SBP trajectory and CVDs. The associations remain significant after additional adjustment of baseline SBP in model 1, and still remain significant for fluctuant and increase group in the fully-adjusted model. Accordingly, we revised the description in statistical analysis (see page 8, lines 160-161) and the notes under Table 2.

4. Given the proportion of loss to follow-up is high, and CV events within this study is self-reported, bias exists where a certain proportion of participants who had developed debilitating or lethal CVD, or died due to other cause, were neglected in the analyses due to the study design.

Answer: In the study cohort, the rate of lost to follow-up ranged from 9.7% in 1991 to 36.4% in 2011. To minimize the bias due to lost to follow-up, we examined the cumulative risk of CVDs, which was defined as ever report of having CVDs during the study period. In the updated analysis, the information on CVDs were available from all of 3600 study participants in the updated analysis. Thus, the bias due to lost to follow-up for the outcome has been minimized in this study.

Minor comments:

1. Abstract: The OR and 95%CI of the "rapid increase" group is 2.10 (0.95, 4.64) which is non-significant. Thus it's not in support of the conclusion that "Rapid increase" patterns of SBP trajectory are associated with higher risk of CVDs.

Answer: The abstract has been revised based on the new results, see pages 2-3.

2. Line 48-51: The authors expressed that, "BP change over time should be accounted in CVD risk estimation; monitoring BP with lifetime estimate is essential." The second sentence makes it hard to understand what the authors wanted to emphasize, the BP monitoring or the BP change over time, thus the sentences should be re-phrased.

Answer: We have revised the sentence, see page 3, lines 48-50.

**Reviewer #2:**

1. The study showed that the “fluctuant” group started with a low SBP (~70 mmHg) in childhood, which was not consistent with the common sense, please explain the reason.

Answer: The SBP in childhood was estimated based on the model parameters. We agree that the low SBP of 70 mmHg is not common in reality. This could be due to the less reliable trajectory pattern in childhood because of the small number of participants in childhood. We have now acknowledged this as a limitation in the discussion (see page 15, lines 274-275).

2. Tables 2 and S2 showed different results between “Rapid increase” group and “Fluctuant” group, which needed to be further explained.

Answer: We thank the reviewer for pointing this out. Now, the results have been updated based on the participants with 3 SBP measurements (n=3600) according to #1 Reviewer’s suggestion. The association between SBP trajectory and CVDs are shown in Table 2 and in Results (page 12, lines 199-207).

3. The table did not show the characteristics of the study population across trajectory groups (n=3750), which were analyzed in this study.

Answer: We have updated the analysis in 3600 participants, which is the final sample with at least 3 SBP measurements and information on CVDs. The characteristics across trajectory groups are shown in Table 1 and Results (see page 9, lines 190-198).

4. Did the events include both recurrent and new cases of CVD?

Answer: We used self-reports of ever diagnosed CVDs as the outcome. The question is worded as follows: “Has a doctor ever given you the diagnosis of myocardial infarction (or stroke)?” Hence outcome included all events of CVDs. To make it clear, we have added the information in the methods section (see page 6, lines 108-109). We also revised the discussion to include the new and recurrent cases of CVDs (see page 15, lines 279-280).
