# Supplementary material for: Life-course blood pressure trajectories and cardiovascular diseases: A population-based cohort study in China
Source: PLoS One. 2020 Oct 21;15(10):e0240804. doi: 10.1371/journal.pone.0240804 (PMC7577482; doi:10.1371/journal.pone.0240804)
Supplement: Attachment — Submitted filename: Response to comments_2020Sept17.docx [file pone.0240804.s007.docx]

**Reviewer #1:**

Three additional major comments should be addressed, on trajectory age range, use of antihypertensives and follow-up lost.

Answer: We thank the reviewer very much for the further comments. We have addressed these three comments, please see the details of revisions as specified below.

Re. authors' reply #1:

Baseline age for majority of participants falls in "adulthood" as illustrated in figure 1 and calculated from Table 1 (95% fall at 33.3±1.96*7.0 = 19.6-47.0 if normally distributed). After 20 years of follow-up, they turned 40-67 years ("midlife" and "late life"). Extreme young and old participants should be removed as they contributed to less than 5% of total population and trajectory at such age range was not reliable due to small sample size and lower-than-lifetime follow-up. Since removal of such participants would not change the result to a significant extent, the study will still offer information on blood pressure trajectory of participants from early-to-mid adulthood to mid-to-late adulthood, who are the major target for primary prevention of hypertension-related disorders.

The authors may partially rebut my comment by providing a table in the next revision of manuscript demonstrating that there is a consistent difference of CVD prevalence across blood pressure trajectories at different baseline age groups (e.g. grouped according to figure 1, baseline age: 9-18, 19-45, 46-60 and 61-70) , or, alternatively downplay the trajectory coverage, i.e. adulthood but not lifetime.
Answer: To address the reviewer’s comments, we have now removed the very young and very old participants by limiting the age range at baseline to 20-50 years (n=3566). We have revised the description of participants in the methods, see page 5. The trajectory pattern of SBP remain unchanged (see Fig 1 and S1 table). In addition, we updated the subsequent analysis on associations between SBP trajectories and cardiovascular diseases, see Table 1, S2-S3 tables and the text in the results (see pages 8-14). In addition, we have updated the abstract (see pages 2-3), main findings (see page 14) and discussion (see pages 15-17).

Interestingly, if the trajectories remain unchanged in the participants at adulthood at baseline after re-analysis, then adding of percentage of cardiovascular disease to the figure (see attachment) and limiting age range from 18-50 (black rectangle) will show us that participants at low level of blood pressure over adulthood had the lowest prevalence of cardiovascular disease and CVD prevalence increases with the increment of blood pressure, which is not surprising. Meanwhile, when limiting age range to 40-70 (red rectangle) then participants with highest CVD prevalence (class 4) had lower blood pressure. Since criteria of CVD were severe but non-lethal, then the result could be explained by the usage of antihypertensives for primary and secondary CVD prevention, that participants at class 2, 4 and 5 had significantly higher proportion of antihypertensive use (28%, 68% and 60%). That could also be confirmed from model 3 of table 2 that the significance nearly diminished after adjustment of antihypertensive treatment. A sensitivity analysis is needed to show if such association was still significant in antihypertensive drug-naïve subjects.

Answer: As the reviewer suggested, we have added the cumulative incidence of CVDs to the figure (see Fig 1). We agree with reviewer that use of antihypertensive medication has an effect on the associations between SBP trajectory and CVDs. Now, we have added a supplementary table on the associations stratified by use of antihypertensive medications, see S2 table. Accordingly, we have added these results (see page 13) and a brief discussion (see page 16).

Re. authors' reply #4:
To minimize the impact of lost of follow-up due to various reasons, the authors should conduct another sensitivity analysis by re-evaluate their findings in those who participated the first and the last follow-up (i.e. 63.6% according to line 89).

Answer: As the reviewer suggested, we have performed the sensitivity analysis among those who were followed from baseline until the last follow-up (n=2210). The results were shown in S3 Table. The associations remained unchanged compared with the main results from total participants. Accordingly, we added a brief discussion on the lost of follow-up, see page 17.

**Reviewer #2:**

Because of the small number of participants in childhood and the aged, the trajectory in childhood and later life shown in the figure might be less reliable. It is suggested that the age span in the analyses could be smaller, making the results more reliable.

Answer: We thank the reviewer for kindly providing further comments. As the reviewer suggested, we have removed the very young and very old participants (n=34) from baseline. The age range at baseline has been limited to 20-50 years (n=3566). We have described this in the methods, see page 5. Accordingly, we have updated the trajectory analysis (see Fig 1 and S1 Table) and subsequent analysis on associations between trajectory groups and cardiovascular diseases (see Table 1). Please also refer to our response to the first comments of Reviewer #1.
